# Cincinnati College of Medicine and Surgery—Dental Department

**Published:** 1892-04

**Authors:** 


CINCINNATI COLLEGE OF MEDICINE AND SURGERY—DENTAL DEPARTMENT.—The commencement exercises of the Dental Department of the Cincinnati College of Medicine and Surgery were held at the Y. M. C. A. Hall, Cincinnati, O., on Wednesday evening, March 16th, 1892.

The exercises were opened with an overture, followed by prayer.

The number of graduates were ten, and the number of matriculates for the session of 1891-'92 were thirty-four.

The degree of D. D. S. was conferred on the following graduates:

Ernest Bragdon, M.D., James F. Clayton, B. Frank Corwin, G. W. Hoffman, James Franklin McCamant, Clifford Edwin Sibbet, William Francis Sloan, John C. Wallace, M.D., Samuel H. Wardle, Fred. G. Williams.

